# A Novel Genetic Sexing Strain of *Anastrepha ludens* for Cost-Effective Sterile Insect Technique Applications: Improved Genetic Stability and Rearing Efficiency

**DOI:** 10.3390/insects12060499

**Published:** 2021-05-27

**Authors:** Edwin Ramírez-Santos, Pedro Rendon, Georgia Gouvi, Antigone Zacharopoulou, Kostas Bourtzis, Carlos Cáceres, Kenneth Bloem

**Affiliations:** 1 Laboratorio El Pino, Programa MOSCAMED, Km 47.5 Carretera a El Salvador, Parque Nacional Laguna El Pino, 06002 Santa Rosa, Guatemala; 2 International Atomic Energy Agency–Technical Cooperation TCLAC, Programa Moscamed/USDA, Guatemala; p.a.rendon-arana@iaea.org; 3 Insect Pest Control Laboratory, Joint FAO/IAEA Centre of Nuclear Techniques in Food and Agriculture, Seibersdorf, A-1400 Vienna, Austria; G.Gouvi@iaea.org (G.G.); K.Bourtzis@iaea.org (K.B.); C.E.Caceres-Barrios@iaea.org (C.C.); 4 Department of Environmental Engineering, University of Patras, 2 Seferi Street, 30100 Agrinio, Greece; 5 Biology Department, University of Patras, 26500 Patras, Greece; zacharop@upatras.gr; 6 Retired, USDA-APHIS-PPQ, Science and Technology, Raleigh, NC 27606, USA; kenneth.bloem@usda.gov

**Keywords:** irradiation, translocation, quality control, insect pest control

## Abstract

**Simple Summary:**

Tephritid flies, including the Mexican fruit fly *Anastrepha ludens*, are key agricultural pests responsible for billions of dollars of damage each year due to the female flies which lay eggs and develop maggots in the fruits of hundreds of species of economically-important plants. Integrated pest management practices such as the sterile insect technique were developed which rely on the mass rearing and release of millions of sterile males of the same pest species in order to suppress the pest reproductive capacity. The presence of females in early bisexual strains often caused high rearing and release costs plus damage to crops by oviposition. Therefore, using classical genetic tools, genetic sexing strains were developed for some fruit flies in order to produce male-only colonies which enhanced the technique efficacy, cost-efficiency and reduced undesirable side-effects such as fruit damage by the mass reared females. In this paper the process for the development and characterization of a new genetic sexing strain (GSS) for *A. ludens* is documented, using the low-dose irradiation and line selection techniques. The new GUA10 GSS, when compared with its predecessor Tapachula-7, promises to further increase the sterile insect technique performance given its higher quality, yield and genetic stability.

**Abstract:**

*Anastrepha ludens* (Loew) is one of the most destructive insect pests damaging several fruits of economic importance. The sterile insect technique (SIT) is used under an area-wide integrated pest management approach, to suppress these pest populations. Mass rearing facilities were initially established to produce sterile males of bi-sexual strains in support of SIT. The first genetic sexing strain (GSS) for *A. ludens*, Tapachula-7, based on pupal color dimorphism, was a key development since the release of males-only significantly increases the SIT efficiency. In this study, we document the development of a novel pupal color-based GSS. Twelve radiation-induced translocation lines were assessed as potential GSS in terms of recombination rates and rearing efficiency at a small scale. The best one, GUA10, was cytogenetically characterized: it was shown to carry a single translocation between the Y chromosome and chromosome 2, which is known to carry the *black pupae* marker. This GSS was further evaluated at medium and large scales regarding its genetic stability, productivity and quality versus Tapachula-7. GUA10 presented better genetic stability, fecundity, fertility, production efficiency, flying ability, and male mating, clear indicators that GUA10 GSS can significantly improve the efficacy and cost-effectiveness of SIT applications against this pest species.

## 1. Introduction

The Mexican fruit fly or mexfly, *Anastrepha ludens* (Loew) Diptera: Tephritidae, is one of the most important agricultural pests in North and Central America due to the damage it produces to more than 40 species of fruit-producing plants of economic importance [1], including mangoes and several species of citrus and deciduous crops. Although the number of potential host plants is not as high as that for the medfly, *Ceratitis capitata* (Wied.) [2], it is still considered a high-risk pest [3]. Countries, where these and other fruit fly pests are present, suffer significant loss of produce and face severe limitations in exports of fruits and vegetables to pest-free countries which represent gourmet markets with premium prices. In addition, some markets accept the reception of fruits and vegetables only from pest-free countries and areas or from areas where the pest is under control and the products are subject to post-harvest treatment, a process that is costly and reduces farmers’ income [4]. In addition, global climate change adds a new dimension to this threat as these tephritid pests expand their geographical range and increase their growth rate in response to global warming [5].

The sterile insect technique (SIT) is a genetic method commonly used as a component of area-wide integrated pest management (AW-IPM) programmes against insect pests and disease vectors. It was originally conceived by Knipling for the control of the screwworm *Cochliomyia hominivorax* (Coquerel) Diptera: Calliphoridae [6,7]. Due to the successful eradication of this species from North and Central America, its development and implementation was expanded in several other insect pest species including several fruit flies of major economic importance [8]. SIT is based on the induction of dominant lethal mutations via ionizing radiation which render the insects sterile. The SIT package also includes the large-scale production of insects in mass rearing facilities and the release of sterile insects in the field in a ratio that guarantees their mating with wild females, thus inducing reproductive sterility and effectively the population control of insect pests such as the tephritid species the Mediterranean fruit fly (medfly), *Ceratitis capitata*, and the Mexican fruit fly (mexfly), *A. ludens*; among other species [9,10,11].

In the initial applications of SIT against the medfly and the mexfly, bisexual strains were used. However, the unnecessary rearing of females significantly increased the costs [12], including the logistics with respect to the rearing, irradiation, transportation and release. These females, though sterile, were still able to produce oviposition damage to fruits. Additionally, much of the sterile sperm was wasted in matings between the sterile males and their female siblings significantly reducing the SIT efficiency. Therefore, the removal of females prior to release is of great importance, both in terms of economics and efficiency in the field [13]. The development of genetic sexing strains (GSS), which allow the separation of males and females or the elimination of females significantly enhanced SIT applications because it allowed the release of virtually 100% males in the field thus reducing the overall cost and avoiding the negative impact related with the release of females [14].

The construction of a GSS requires two components: (a) the availability of recessive morphological or conditional lethal mutations and (b) the linkage of wild type alleles of the marker genes to the male determining region, which is usually achieved with radiation-induced reciprocal translocation between the sex chromosome Y and the autosome that carry the wild-type alleles of the selectable markers T(Y-A) and allows the physical or mechanical sex separation at the earliest possible developmental stage [15]. In tephritids, the first GSS was developed for *C. capitata* using as a selectable marker the pupal color, which is determined by the *white pupae (wp)* gene, with heterozygous males emerging from brown pupae and homozygous females emerging from white pupae [16]. The sex separation was carried out by optical and mechanical means [17,18]. Although this made possible the release of sterile male-only in the target areas, the sexing mechanism was not ideal since the separation only occurred during the pupal stage thus demanding the rearing of female larvae and increasing the overall costs and logistics [13]. The second generation of the medfly GSS was developed by the introduction of a *temperature sensitive lethal* (*tsl*) gene, closely linked with the *white pupae (wp)* gene, [19], which allowed the elimination of the homozygous *tsl* females at the embryonic stage [20] via their exposure at elevated temperatures (i.e., 32–34 °C). The early elimination of females improved the efficiency and lowered the costs of mass rearing and, in addition, enhanced the overall efficacy and cost-effectiveness of SIT applications [21]. In the case of the mexfly, a pupal color-based GSS, Tapachula-7, has been developed using the same approach as in the medfly first-generation sexing strains, but with a different selectable marker the *black pupae (bp)* gene, which is located on polytene chromosome III (mitotic chromosome 2). A radiation-induced T(Y-2) reciprocal translocation linked the wild type allele of this gene to the male determining region and facilitated the sex separation, with heterozygous males emerging from brown pupae and homozygous females emerging from black pupae [22,23]. 

A critical aspect for all GSS developed following the aforementioned approach is the genetic integrity and stability of the strains due to different genetic recombination events. Such phenomena have been well studied in puparium-colored GSS. It has been shown that unexpected progeny, for example recombinants (adults emerging from the wrong pupae color), can start being accumulated thus putting at risk the genetic integrity of the GSS and reversing its genetic sexing mechanism [19]. The recombination rates depend on several factors including the distance between the selectable marker and the translocation breakpoint as well as the relative position of the recombination point to the centromere, with translocations closer to the centromere being more stable [16,21].

The mass rearing of a GSS usually involves the implementation of a filter rearing system (FRS) [20] and allows the maintenance of a steady genetic sexing mechanism by means of the selection of genetically stable individuals in base colonies, which are amplified in number during the next generations. For the mass rearing of *A. ludens* GSS, the FRS starts with a small mother colony named the Filter, where recombinants are manually eliminated. From the filter colony, the offspring is amplified in two or three additional generations until the last colony can provide sufficient eggs for the production of male-only pupae and adults for field releases. 

In an effort to develop steady GSS for an SIT target species, therefore, it is important to induce and characterize a number of T(Y:A) translocations in order to select the one(s) with the best genetic stability, production profile and overall quality. In the present study, our aim was to induce and characterize new (Y:2) reciprocal translocations in the major agricultural insect pest species, *A. ludens*, which would result in novel GSS with improved genetic stability and better performance with respect to their productivity and mating performance. Once a better line (later named as GUA10) was identified and further characterized as a potential new GSS, its performance was also compared to that of the original *A. ludens* GSS (known as Tapachula-7 or TAP7).

## 2. Materials and Methods

Site of the experiment and source of the insects. This study was conducted at the San Miguel Petapa (SMP) laboratory from the Moscamed Program, 15 km to the south of Guatemala City (N 14°29’2”, W 90°36´53”) in areas dedicated for the mass rearing of *A. ludens*. The rearing conditions were: temperature 25 ± 2 °C, relative humidity 65 ± 5% and photoperiod of 12:12 h. 

Fruit fly lines. Wild Mexican fruit flies were obtained from field collections of Rutaceae, matasano (fruits *Casimiroa tetrameria* Millsp). About 2000 flies were adapted to laboratory conditions for 10 generations before they were used in experiments. The original black pupae GSS Tapachula-7 (abbreviated as TAP7 in this study) was kindly provided by the Moscafrut Program at Metapa de Dominguez, Chiapas, México [22,23].

Rearing conditions. Larval diet used for maintenance of all colonies was a mixture of sugar, torula yeast, corn flour, sodium benzoate, water and corn cob, as described previously [24]. Adult flies were fed on a diet prepared by mixing sucrose and hydrolyzed protein (MP Biomedicals, LLC, Santa Ana, California, EUA) in a 3:1 proportion. Additionally, water was provided through plastic containers. 

Induction and isolation of new GSS lines. Mature wild pupae, 24 h before adult emergence, were exposed to 30 Gy of gamma irradiation by using a cesium 137 irradiator (Isomedix Inc. Husman model 521 series 004 irradiator, 07981 Whippany, NJ, USA). About 3000 pupae were irradiated and then after adult emergence and knockdown with nitrogen gas, around 1000 males were selected, thus representing the parental (P) generation. Irradiated males were crossed en masse with homozygous (bp/bp) black pupae females at 1:2 (male:female) ratio. Two hundred fifty (F1) males were randomly selected and individually backcrossed with homozygous black pupae females. In the next generation (F2), families that produced males from brown pupae and females from black pupae were selected as new potential chromosomal translocations between chromosome Y and autosome 2, which carries the *black pupae* (*bp*) gene.

Fecundity and fertility assessment. The comparison of fecundity (eggs per female per day) and fertility (% egg hatch) in GUA10 and TAP7 strains was conducted under two different stress conditions: high stress conditions by using the standard mass production egging cages [25] and low stress conditions by placing 25 ♀ × 25 ♂ in a 1-L plastic container with a black membrane, ethylene vinyl acetate (Foamy) of 1.5 mm thickness on one side, as an oviposition surface. For high stress conditions, 1300 ♂ × 2700 ♀ were placed in rectangular egg-producing cages (240 × 270 × 270 mm^3^) with a similar membrane on one side, as an oviposition surface. The total eggs per cage collected in 15 consecutive days were used to estimate fecundity while fertility was determined five days after the egg laying based on the hatch rate of a sample of 10,000 eggs collected during the same period.

Genetic stability and productivity of new GSS lines. The assessment of performance of the new GSS (translocation) lines including the original TAP7 GSS, which was used as reference line, was carried at small rearing conditions. The small-scale consisted of seeding units of 1000 eggs for each of the lines which were placed on top of 85 g of larval diet in 100 mm × 15 mm Petri dishes. Twelve translocation lines were tested at small scale for 10 generations to assess their genetic stability (recombination rate, e.g., number of females that emerge from brown pupae or males that emerge from black pupae), yield (egg to pupae conversion rate) and their overall production and quality profiles. Table 1 shows that at generation F10 the most promising line, GUA10, was selected for continuous assessment under small scale conditions in respect to (a) genetic stability (recombination rate) continuously until generation 28 and (b) rearing performance (egg to pupa conversion rate) for eight out of the first 27 generations. Based on the initial encouraging results, GUA10 was later assessed under medium- and large-scale rearing conditions with respect to the following traits: a) fecundity (number of eggs per female), b) fertility (percent hatching and/or egg to pupae or adult conversion rates), and c) genetic stability (recombination rate) and overall mass rearing performance. For the medium-scale assessment, 60 plastic containers (250 × 150 × 60 mm^3^) were used each containing 0.6 mL of eggs (~18,000 eggs/mL, 10,800 eggs per container) per kg of larval diet. The assessment was repeated three times in different days. Yield was determined by counting the total number of pupae recovered and estimating the egg to pupae conversion rate (E:P) by dividing the total number of pupae recovered by the number of eggs seeded. For the large-scale assessment, 220 fiberglass trays (770 × 400 × 70 mm) were used and each tray contained 4.8 kg of larval diet with 2.8 mL of eggs (~61,600 eggs/tray). Yield was measured in two ways: (a) as larval recovery (LR) which is defined as the calculation of the volume (liters) of larvae collected per kg of diet, and (b) as yield in million pupae per ton of diet (M/ton), which was estimated by dividing the total number of pupae collected by the amount of diet. For medium scale, the number of replicates to estimate yield was *n* = 87 for GUA10 and *n* = 66 for TAP7 while at large scale it was *n* = 22 for GUA10 and *n* = 83 for TAP7. The total number of pupae produced was divided into two equal batches, one of them was irradiated and compared to the other one, the non-irradiated batch, in order to document potential effects of irradiation on the quality of the translocation lines studied. The experiment was repeated three times in different days. At the end, additional laboratory and field tests were carried out as described below in order to select the best strain.

Quality Control Laboratory tests. Quality was evaluated according to the QC Manual V6.0 [26] by assessing the following parameters for a given number of replicates (*n*): a) pupae weight (size), b) percentage emergence and flight ability. For the genetic stability (recombination rate) of the lines studied, samples of 100 mL pupae from large rearing scale were examined to determine the sex of the insect emerging from brown or black pupae and their quality. Flight ability was determined for both strains, GUA10 and TAP7, for males (*n* = 29) and females (*n* = 27). The recombination rate was determined for both GUA10 (*n* = 62) and TAP7 (*n* = 52) strains.

Irradiation dose response curve for GUA10. Under hypoxia conditions, 60 mL of GUA10 brown pupae (males) were irradiated at 48 hr before adult emergence [27], with a dose of 0 (control), 20, 40, 60 and 80 Gy. Males of each irradiation dose treatment were kept individually in 300 × 300 × 300 mm plexiglass cages. Once the irradiated males reached sexual maturity (10 days after emergence), they were crossed with fertile mature wild virgin females by setting single pair crosses in small cages. For each treatment, five replicates were set up. Eggs produced by the females were collected for 10 consecutive days (for a total number (*n*) of 50 samples/treatment) from day 3 to 12 according to the QC Manual V6.0 [26]. After a 7-day incubation period, the fertility was determined by recording the number of newly hatched larvae; the percentage of induction of sterility was calculated as follows: (100%–percentage of fertility).

Field cage tests. Field tests were carried out in a 600 m^2^ area within the SMP fields using 0.5 mesh plastic net cages. For the mating and competitiveness tests, circular cages (CC) of 3000 mm in diameter × 2000 mm tall were used while rectangular cages (RC) of 100 × 100 × 140 mm for the longevity tests [26]. Inside each of the CC and RC cages, a young coffee plant (*Coffea arabica* L.) was enclosed. The external temperature was between 24 and 28 °C, the relative humidity between 55% and 70%, and the natural illumination between 600 and 3600 lux. For the field longevity tests under stress conditions (without water and food), 100 GUA10 or TAP7 males were introduced in RC cages. The longevity tests were carried out for each of the four representative pupae sizes (size 4 = 16 mg ± 1 mg; size 5 = 18 mg ± 1 mg; size 6 = 20 mg ± 1 mg; size 7 = 22 mg ± 1 mg). Every 12 h, dead males were removed and counted until reaching 100% mortality. Five to six replicates were carried out for each strain and pupae size group. Two parameters were recorded: (a) the cumulative mortality curve and (b) mean life (hours to reach 50% mortality).

For the field mating tests, TAP7 and GUA10 male pupae were irradiated at 36 hr before emergence at an irradiation dose of 80 Gy (= 8 Krad). Non-irradiated wild males were used as reference. Emerged males were kept in plexiglass cages (300 × 300 × 400 mm) for 10 days until they reached sexual maturity and then 50 males of each group were released in CC cages in order to compete, according to the treatment, for mating with 50 wild and virgin fertile females. Five treatments were evaluated: (a) GUA10 males vs. wild males, (b) TAP7 males vs. wild males, (c) GUA10 males vs. TAP7 males vs. wild males, and (d) as control, two additional treatments were included to measure mating competitiveness of wild males vs. wild females; one in a 2:1 ratio (male: female) as a control for treatments “a” and “b”, the other in a 3: 1 ratio (male: female) as a control for treatment “c”. With a total of four replicates in time, the total number of replicates/treatment was *n* = 20 for treatment c, *n* = 11 for treatment d and *n* = 22 for each of the other treatments. The mating competitiveness in each treatment was assessed by determining the proportion of matings and two sterility indexes: (a) the relative sterility index (RSI) [26] and (b) the relative mating performance index (RMPI) that measures the competition of two different male-types (in this case, GUA10 and TAP7) within the same cage, against wild males. This is an adaptation of RSI that is applied when there is only one kind of laboratory male in competition with wild males. The formula used for calculating RMPI, for GUA10 and TAP7 respectively, is as follows: GUA10 RMPI = GUA10W/WW + GUA10W + TAP7W and TAP7 RMPI = TAP7W/WW + GUA10W + TAP7W. The RMPI is a measure of the propensity of sterile males to mate with wild females, with values ranking from −1 to +1. A value of −1 indicates that all matings were carried out by wild males, while a value of +1 indicates that all matings were carried out by sterile males. Zero indicates that males from both populations participated equally in matings. Field tests were conducted as recommended [26].

Cytogenetic analysis. Third-instar larvae were used for polytene chromosome preparations as described in [28,29]. The dissection of the salivary glands was carried out in 45% acetic acid and they were immediately placed in 1N HCl for 1 min. The fixation of the chromosomes was performed in fixative solution (3:2:1; acetic acid/H_2_O/lactic acid) for three to five minutes, until they became transparent. The chromosomes were then stained in a drop of lactoacetic orcein for approximately 10 minutes. The slipover of the stain was removed by washing the preparations in the fixative solution. After the squashing, the preparations were observed and analysed at 100× oil objective (1000× magnification) with reference to the salivary gland chromosome maps [30].

Statistical analysis. Data sets were checked for normality by means of a Kolmogorov–Smirnov or Shapiro–Wilk test, and homoscedasticity (equal variances). Analysis of the variables was performed through a 1-way or 2-way analysis of variance (ANOVA) with different number of replicates per treatment. When differences were significant, they were further separated by means of a Tukey test. For the percentage of egg hatching and biological dosimetry, an arcsin transformation was applied; however, as significance did not change, the original non-transformed data are shown in the results. For the rest of the variables, no transformation was needed. In all tests, a significance level was calculated. The software used was InfoStat/P [31]. All of the original data can be found in the supplementary file.

## 3. Results

### 3.1. Induction of New Translocations and Selection of New Genetic Sexing Strain (GSS)

Two hundred and fifty lines (families) were screened for the presence of translocations, evidenced as production of males from brown pupae and females from black pupae. Twelve of them were selected as potential new GSS for further assessment with respect to their productivity and genetic stability for 10 generations. As shown in Table 1, five families were discarded due to high recombination rates, six lines were discarded due to low productivity, and only one (named GUA10) exhibited satisfactory productivity and genetic stability and was selected for further evaluation and comparison with the previously developed GSS, TAP7.

### 3.2. Fecundity and Fertility

As shown in Table 2, the GUA10 and TAP7 GSS differed significantly in respect to both fecundity and fertility, with the GUA10 strain being superior to TAP7. Regarding the fecundity, the average number of eggs per female per day was significantly higher for GUA10 when compared to TAP7 under low stress (F = 155.13, *p* < 0.0001) but not in high stress conditions (F = 3.49, *p* = 0.0715). With regard to the fertility, the average % egg hatch rate was significantly higher for GUA10 as compared to TAP7, under low stress (F = 141.36, *p* < 0.0001) and high stress conditions (F = 59.47, *p* < 0.0001).

### 3.3. Genetic Stability and Productivity of the Selected GSS GUA10

**Figure 1:**
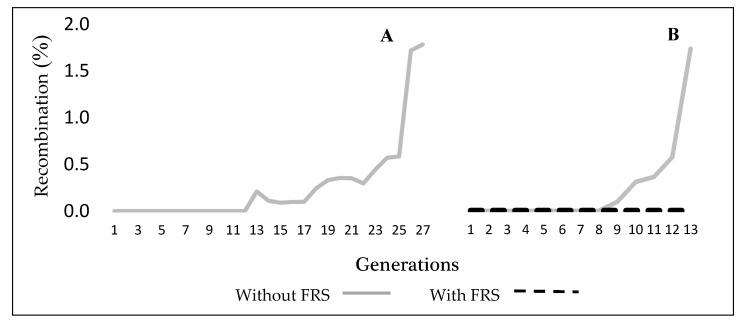
Recombination rate in *Anastrepha ludens* GUA10 GSS. (**A**). recombinants accumulated for 27 generations at small rearing scale. (**B**). recombinants accumulated for 13 generations at large rearing scale.

As shown in Figure 1, the GUA10 line presented very low recombination rates for almost 27 generations (about 3 years), thus confirming its genetic stability. It is worth noting that no female recombinants (female emerging from brown pupae) were detected under small scale rearing conditions. Male recombinants emerging from black pupae were detected at about 1.7% rate at generation 27 and 13 during small- and large-scale rearing conditions, respectively (Figure 1A,B). At large-scale rearing conditions, the genetic recombination was closely monitored with and without filtering (Figure 1B); the FRS was able to keep recombination rate at zero.

**Figure 2:**
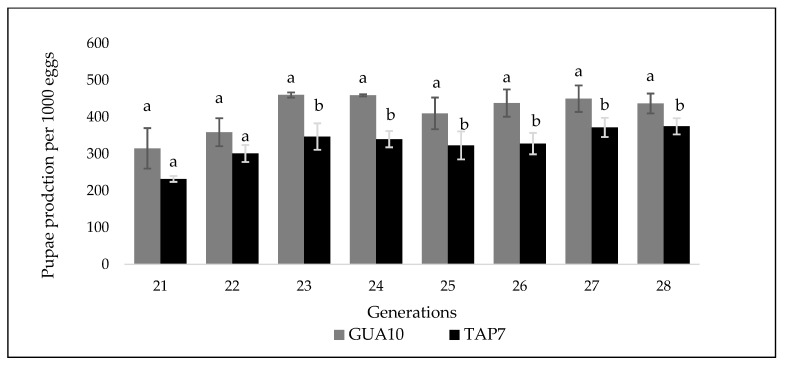
Egg to pupae conversion rate of *Anastrepha ludens* GUA10 and TAP7 GSS for eight generations at small scale. Differences are highly significant for each strain and generation. Pairs of means (± SD) with the same letter are not significantly different.

The GUA10 GSS was also assessed in respect to its productivity under different conditions and in comparison with the TAP7 GSS. As shown in Figure 2, GUA10 exhibited higher (F = 13.20, *p* = 0.0027) egg to pupae conversion rates than TAP7 for eight continuous generations under small-scale rearing conditions. Also as shown in Table 3, under both medium (F = 53.73, *p* < 0.0001) and large-scale (F = 12.79, *p* = 0.0005) rearing conditions, GUA10 GSS exhibited significantly higher larval recovery (LR), egg to pupae (E:P) conversion efficiency (F = 43.94, *p* < 0.0001; F = 16.51, *p* < 0.0001, respectively) and production in millions of pupae per ton of larval diet (M/ton) (F = 92.97, *p* < 0.0001; F = 12.8, *p* = 0.0005, respectively) thus producing, on average, about 29% more pupae under large (mass rearing) scale.

### 3.4. Quality Control Analysis under Laboratory Conditions

As shown in Table 4, at large scale (mass rearing) the GUA10 and TAP7 GSS differed significantly in respect to their quality, with the GUA10 GSS presenting higher adult emergence and flight ability rates, for males (F = 243.17, *p* < 0.0001; F = 119.56, *p* < 0.0001, respectively) and females (F = 89.33, *p* < 0.0001; F = 11.42, *p* = 0.0013, respectively), and lower recombination rates in brown color pupae (F = 36.26, *p* < 0.0001). The size of the GUA10 GSS pupae is larger than that of the TAP7 GSS pupae, but the size distribution is very similar to that of the wild (W) flies. In the wild strain, 90% of the pupae are within sizes 5 to 8 (18–23 mg).

### 3.5. Irradiation doses Response Curve for GUA10

**Figure 3:**
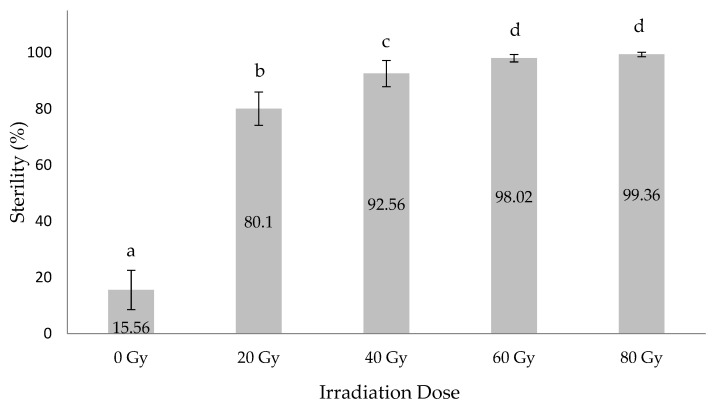
Induced sterility (%) of the GUA10 GSS males irradiated at different doses. Means (± SD) with different letters are significantly different.

As shown in Figure 3, the GUA10 GSS males presented a sterility induction pattern similar to that previously reported for the TAP7 GSS when irradiated at doses between 0 and 80 Gy [32]. Statistical analysis suggsted that there was no significant difference in the levels of induced sterility between irradiation at 60 Gy and 80 Gy (F = 2892.88, *p* < 0.0001); however, the application of an 80 Gy irradiation dose induced almost complete male reproductive sterility reaching an average level of 99.36%.

### 3.6. Field Cage Tests

**Figure 4:**
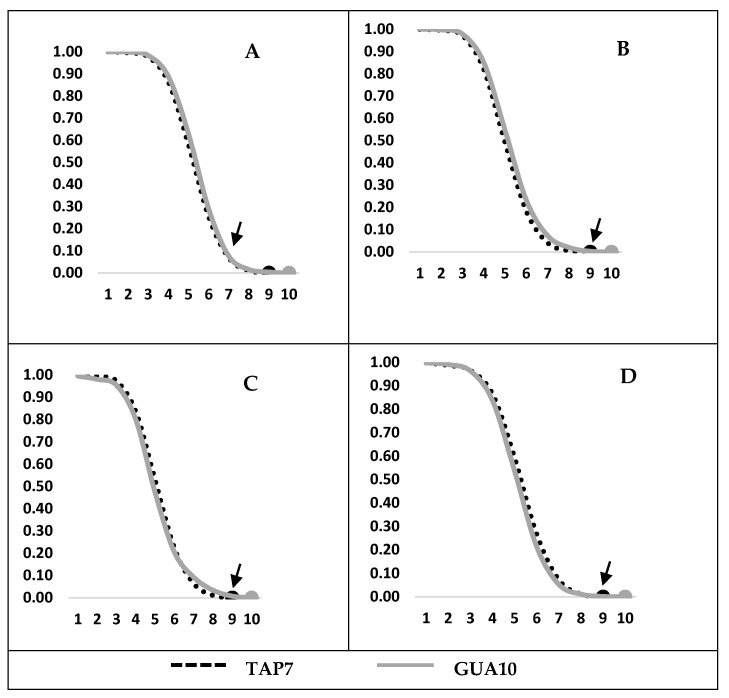
(**A**–**D**) shows survival rate for pupae size 4, 5, 6 and 7, respectively.

There is no significant difference in the longevity of GUA10 and TAP7 GSS males under stress conditions (without food or water). Irrespective of the pupal size, the mean life was high, between 119 and 129 h. Figure 4A–D confirm that the survival rate (lx, equivalent to the % of live adults, expressed from 0 to 1) for each pupae size between 4 (17 mg) to 7 (22 mg) is basically the same for both strains with one minor difference: TAP7 males reach 100% mortality at about day 9, one day earlier than GUA10 males.

As shown in Table 5, the RMPI, which ranges from −1 to +1, was significantly (F = 5.87, *p* = 0.0198) higher for GUA10 (0.40) compared to TAP7 (0.32) when wild males competed together with GUA10 and TAP7 GSS males for mating with wild females. It is worth noting that 51.27% successful matings were recorded in the control group (w♂ × w♀) ratio 3♂:1♀ while, when GUA10, TAP7 and wild males competed together for wild females (ratio 3♂:1♀), 19.10%, 16.30% and 14.70% successful matings were registered respectively (50.10% in total) (F = 18.07, *p* < 0.0001). In the cages where GUA10 and wild males competed for wild females (ratio 2♂:1♀), 30.73% and 16.09% successful matings were recorded respectively (46.82% in total), a difference which is statistically significant (F = 9.25, *p* = 0.0004). In cages where TAP7 and wild males competed for wild females (ratio 2♂:1♀), TAP7 registered 22.73% matings and wild males 20.55% (43.28% in total), and there was no significant difference between TAP7 and wild males (F = 0.30, *p* = 0.5877). It is worth noting that 41.45% successful matings were recorded in the control group (w♂ × w♀) ratio 2♂:1♀. The relative sterility index (RSI) was significantly higher (F = 9.71, *p* = 0.0033) for GUA10 males (0.68) compared to TAP7 males (0.54) when each competed against wild males for mating with wild females in independent cages. These results show that both GUA10 or TAP7 mass-reared males were engaged in more than 50% of the total matings compared with wild females.

### 3.7. Cytogenetic Analysis of GUA10 Translocation

**Figure 5:**
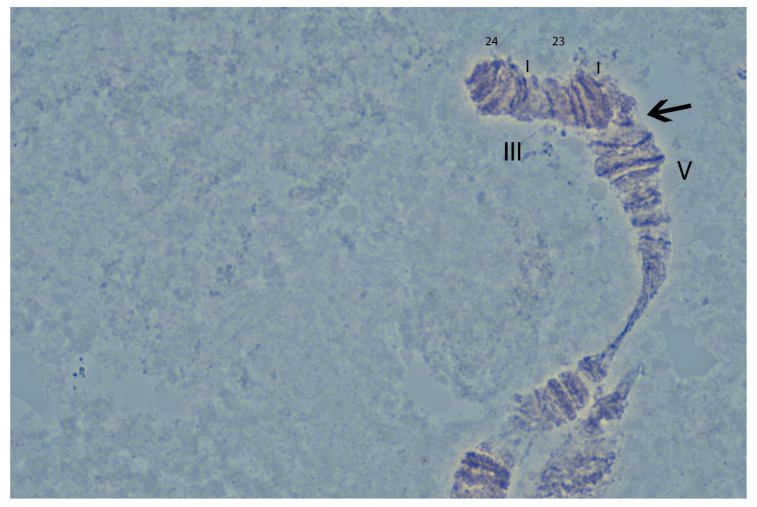
The region of polytene chromosome 3 involved in the Y-III translocation in the *Anastrepha ludens* GUA10 genetic sexing strain (100× magnification). The arrow indicates the translocation break point.

The cytogenetic analysis was challenging because of the capacity of the *A. ludens* chromosomes for inter- and intraectopic pairing, curling and wringing, as well as their frequent breaking, observations that were previously reported for *A. ludens* Tapachula-7 GSS. Based on our analysis, and similarly to the *A. ludens* Tapachula-7 (TAP7) GSS [22], the breakpoint was identified in the ectopic pairing between the polytene elements 2 and 5, albeit at a slightly different position as shown in Figure 5.

## 4. Discussion

The availability of genetically stable and high-quality genetic sexing strains can significantly enhance the efficiency and cost-effectiveness of SIT applications against a target pest population [33]. Given that a number of factors may affect the rearing efficiency, genetic stability and biological quality of a GSS [19], in the present study, we developed several irradiation-induced translocations in *A. ludens*, and 12 of them were selected for the development and further evaluation of pupal color-based GSS. Based on their genetic stability and rearing efficiency under small scale conditions, only one of them, GUA10, was selected for comparative evaluation with a previously developed GSS, TAP7 [22]. The results clearly indicated that the *A. ludens* GUA10 GSS presents higher genetic stability, egg fecundity and fertility, egg to pupae efficiency, production of larvae and pupae per ton of larval diet, quality and mating performance, and the males irradiated with a dose of 80 Gy can induce almost complete sterility. It is important to note that the better performance of the GUA10 GSS was evident under small-, medium- or large-scale rearing conditions suggesting that it has the potential to improve the efficacy of SIT applications against this major agricultural insect pest species.

One of the key characteristics of the GUA10 GSS is its noteworthy genetic stability exhibiting 0% female recombinants (emerging from brown pupae) at both small and large scale rearing conditions and a very low male recombination rate (0–1.7%), which reaches its maximum value only at generation 27 and 13 under small- and large-scale rearing conditions, respectively. Overall, the recombination rate in GUA10 GSS was found to be significantly lower than that recorded in TAP7 GSS (the latter reaches at 12.6% under large-scale rearing conditions). This may be due to the different position of the translocation break point in GUA10 and its relative distance from the *bp* locus. Indeed, as shown by the cytogenetic analysis in Figure 5, the breakpoint in GUA10 is on band 24 of polytene chromosome III while the respective breakpoint in TAP7 was reported on band 25 on the same polytene element [22].

In addition, fecundity, fertility as well as the productivity as expressed by the egg-to-pupae conversion rate were significantly higher in GUA10 compared to TAP7. This is of paramount importance, particularly in large scale operational programmes, because it can result to increased productivity and, consequently, significant reduction in the mass rearing costs. This data confirmed previous observations on *C. capitata* VIENNA GSS [21] that a large number of translocation lines need to be developed and tested in order to select the best one(s) in respect to genetic stability and rearing efficiency. It is interesting to note, however, that there was no major difference between the two GSS, GUA10 and TAP7, in respect to the longevity or the irradiation dose (80 Gy) required to induce sterility levels more than 99%, a level that complies with the standards for SIT applications against mexfly [26].

One of the most important findings of the present study was that males of the GUA10 GSS showed better mating indexes (RMPI and RSI) compared to males of the TAP7 GSS by using established tests [26,32,34]. This is of major applied significance because it suggests that: (a) GUA10 males will present a better sexual performance in the field, (b) under field conditions, wild females will show preference for mass-reared males over wild males which is desirable in any area-wide pest control program and, (c) lower numbers of GUA10 adult sterile males will be needed in the field in order to reach an overflooding ratio that guarantees the effective suppression of the target wild population. In turn, this would mean that the GUA10 GSS males have the potential to induce higher levels of sterility in the field and, therefore, the target wild population can be suppressed faster and more cost-effectively.

Considering the good performance of GUA10 GSS, producing 29% more flies at large scale while keeping costs constant, this clearly suggests that the overall cost efficiency in mass rearing may be proportionally improved. Moreover, combining the increased fecundity (+28%) and fertility (+21%) of GUA10 GSS females at large scale, the economic impact of these enhanced traits means an overall increase 55% in production efficiency (1.28 × 1.21 = 1.55%), indicating that direct mass-rearing costs might reduce proportionally in terms of production areas, equipment, larval/adult diets, personnel and energy.

There is a direct correlation between the quality and fitness of a mass reared insect strain for sterile-insect technique applications and the success of an area-wide pest control program [35]. The results of this study suggest that the generation of new GSS using radiation and classical genetics approaches and the proper characterization of the translocation lines can result in the selection of the best possible GSS. Indeed, our results suggest that, even though both GSS use the females originating from the same black pupae mutant line, the translocation GUA 10 resulted to a GSS with better genetic stability, higher productivity, better flight ability and mating competitiveness than TAP7. All these characteristics were evident even at large-scale rearing conditions strongly suggesting that the GUA10 GSS can significantly improve both the efficacy and the cost-effectiveness of the SIT applications against the mexfly, *A. ludens*.

## 5. Conclusions

The results of this study suggest that the generation of a new *A. ludens* GSS using classical genetics and proper line-selection approaches was successful. The evaluation of the new line, GUA10, clearly indicated that this line can significantly enhance the SIT and can have a positive impact on the overall performance of the large-scale operational programme against the mexfly. The GUA10 GSS presented increased productivity by means of improving: (a) pupae weight which is associated with better longevity and overall insect quality, (b) emergence and flight ability which means less pupae need to be produced to achieve the desired number of males needed for a given overflooding ratio that guarantees the population suppression of the target wild populations; longevity which determines the number of flight-releases per week needed to keep a steady number of sterile males in the field and (c) acceptable mating indices guarantee the induction sterility in the field. In addition, the GUA10 GSS presented significantly higher genetic stability which is important for stable production of male-only pupae. Taken together, the high quality could reduce the overall costs of the SIT program.

## Figures and Tables

**Table 1:** Genetic stability (% recombination ± standard error (SE)) and average productivity (egg to pupae conversion rate ± SE) measured at generation F10 for 12 selected genetic sexing strains (GSS) of *Anastrepha ludens* (Loew). Eggs per replicate = 1000.

Family	*n*	% Recombinants (♀ in Brown Pupae)	% Recombinants (♂ in Black Pupae)	Egg to Pupae Conversion Rate (E:P)
197	6	25.03 ^a^ ± 1.397	22.19 ^a^ ± 1.677	0.36 ^c^ ± 0.005 ×
109	17	0.28 ^c^ ± 0.153	0.20 ^c^ ± 0.110	0.31 ^c^ ± 0.019 ×
48	6	5.30 ^b^ ± 2.672	9.97 ^b^ ± 4.538	0.30 ^c^ ± 0.028 ×
45	14	0.27 ^c^ ± 0.220	0.00 ^c^ ± 0.000	0.34 ^c^ ± 0.016 ×
9	17	2.80 ^b^ ± 1.569	0.97 ^c^ ± 0.533	0.29 ^c^ ± 0.019 ×
87	17	0.00 ^c^ ± 0.000	0.00 ^c^ ± 0.000	0.34 ^c^ ± 0.012 ××
125	17	0.00 ^c^ ± 0.000	0.00 ^c^ ± 0.000	0.33 ^c^ ± 0.019 ××
116	17	0.00 ^c^ ± 0.000	0.00 ^c^ ± 0.000	0.32 ^c^ ± 0.013 ××
154	17	0.00 ^c^ ± 0.000	0.00 ^c^ ± 0.000	0.32 ^c^ ± 0.014 ××
103	17	0.00 ^c^ ± 0.000	0.00 ^c^ ± 0.000	0.32 ^c^ ± 0.025 ××
66	17	0.00 ^c^ ± 0.000	0.00 ^c^ ± 0.000	0.31 ^c^ ± 0.017 ××
10	17	0.00 ^c^ ± 0.000	0.00 ^c^ ± 0.000	0.41 ^b^ ± 0.016 ×××
TAP7 (Control)	17	3.02 ^b^ ± 0.725	0.56 ^c^ ± 0.202	0.32 ^c^ ± 0.015
Wild (Reference)	13	–	–	0.74 ^a^ ± 0.029

Pairs of means with different letters are significantly different. *n* = number of replicates, × Discarded due to high recombination rate (>0), ×× Discarded due to low productivity (E:P < 0.4), ××× Selected line (GUA10).

**Table 2:** Fecundity (average eggs per female per day ± SE) and fertility (% egg hatch ± SE) for GUA10 and TAP7 GSS pairs.

Cross			Low Density (without Stress)				High Density (with Stress)			
Male	×	Female	*n*	Eggs/♀/Day	*n*	% Egg Hatch	*n*	Eggs/♀/Day	*n*	% Egg Hatch
GUA10	×	GUA10	90	49.50 ^a^± 1.33	105	86.92 ^a^± 0.71	14	29.14 ^a^± 2.30	10	85.54 ^a^± 1.33
TAP7	×	TAP7	82	25.57 ^b^± 1.39	96	74.68 ^b^± 0.74	12	22.75 ^a^± 2.49	9	70.67 ^b^± 1.40

Pairs of means with different letters are significantly different. *n* = number of replicates.

**Table 3:** Production profile in terms of larval recovery per kg of diet (mean ± SE), egg to pupae conversion efficiency (mean ± SE) and production in millions of pupae per ton of diet (mean ± SE) for GUA10 and TAP7 GSS under Medium- and Large-scale rearing conditions.

Scale	Strain	Larval Recovery	Egg: Pupae Conversion Efficiency	Millions of Pupae/ton of Larval Diet
Medium	GUA10	0.18 ^a^ ± 0.003	0.26 ^a^ ± 0.01	4.17 ^a^ ± 0.102
	TAP7	0.13 ^b^ ± 0.007	0.17 ^b^ ± 0.008	2.81 ^b^ ± 0.090
Large	GUA10	0.14 ^a^ ± 0.003	0.28 ^a^ ± 0.005	3.60 ^a^ ± 0.081
	TAP7	0.11 ^b^ ± 0.005	0.21 ^b^ ± 0.009	2.78 ^b^ ± 0.117

Pairs of means with different letters are significantly different.

**Table 4:** Quality control analysis (± SE) of the GUA10 and TAP7 GSS in respect to their adult emergence, flight ability, and recombination rates. Pupae and adult samples from large (mass rearing) scale.

	Males (Brown Pupae)		Females (Black Pupae)		Recombinants
Strain	Emergence	Flyers	Emergence	Flyers	(♀ in Brown Pupae)
GUA10	93.36 ^a^ ± 0.652	89.62 ^a^± 0.924	89.54 ^a^ ± 0.618	81.65 ^a^ ± 1.033	1.25 ^a^ ± 0.199
TAP7	78.67 ^b^ ± 0.681	78.76 ^b^ ± 0.364	81.59 ^b^ ± 0.571	77.37 ^b^ ± 0.669	12.65 ^b^ ± 1.740

Means with different letters within a column are significantly different.

**Table 5:** Percentage of matings (± SE) and mating indexes (± SE) of GUA10 vs TAP7 under field conditions.

	Cross							
	(♂GUA10 × ♂TAP7 × ♂w) × ♀w			(♂GUA10 × ♂w) × ♀w			(♂TAP7 × ♂w) × ♀w	
Male	*n*	% Matings ± SE	RMPI	*n*	% Matings ± SE	RSI	*n*	% Matings ± SE
GUA10	20	19.10 ^b^ ± 2.32	0.40 ^a^ ± 0.03	22	30.73 ^a^ ± 3.29	0.68 ^a^ ± 0.03		
TAP7	20	16.30 ^b^ ± 2.27	0.32 ^b^ ± 0.02			0.54 ^b^ ± 0.02	22	22.73 ^b^ ± 2.56
W	20	14.70 ^b^ ± 2.26		22	16.09 ^b^ ± 2.82		22	20.55 ^b^ ± 3.07
Control	11	51.27 ^a^ ± 8.55		11	41.45 ^a^ ± 7.37		11	41.45 ^a^ ± 7.37

Means with different letters are significantly different. *n* = number of replicates (cages with a given male:female ratio). RMPI = Relative Mating Performance Index. RSI = Relative Sterility Index. W = wild population. Control = (♂w × ♀w).

## Data Availability

The data presented in this study are available in supplementary file S1.

## Supplementary Materials

The following are available online at https://www.mdpi.com/article/10.3390/insects12060499/s1, File S1: Raw data.

## References

[1] APHIS (2016). Anastrepha Ludens, Mexican Fruit Fly Host List. https://www.aphis.usda.gov/plant_health/plant_pest_info/fruit_flies/downloads/host-lists/mexfly-host-list.pdf.

[2] Copeland R.S., Wharton R.A., Luke Q., Meyer M. (2002). Indigenous hosts of Ceratitis capitata (Diptera: Tephritidae) in Kenya. Ann. Ent. Soc. Am..

[3] Molina-Nery M., Ruiz-Montoya L., Zepeda-Cisneros C., Liedo P. (2014). Genetic structure of populations of *Anastrepha ludens* (Diptera: Tephritidae) in Mexico. Fla. Entomol..

[4] Hernández E., Aceituno-Medina M., Toledo J., Bravo B., Caro-Corrales J., Montoya P., Mangan R. (2017). The Effects of a Modified Hot Water Treatment on *Anastrepha ludens* (Diptera: Tephritidae)-Infested Mango. J. Econ. Entomol..

[5] Aluja M., Birke A., Ceymann M., Guillén M., Arrigoni E., Baumgartner D., Pascacio-Villafán C., Samietz J. (2014). Agroecosystem resilience to an invasive insect species that could expand its geographical range in response to global climate change. Agric. Ecosist. Environ..

[6] Knipling E.F. (1959). Sterile-male method of population control. Science.

[7] Knipling E.F., Graham O.H. (1985). Sterile insect technique as a screwworm control measure: The concept and its development. Symposium on Eradication of the Screwworm from the United States and Mexico.

[8] Klassen W., Curtis C.F., Dyck V.A., Hendrichs J., Robinson A. (2005). History of the Sterile Insect Technique. Sterile Insect Technique.

[9] Dominiak B.C., Westcott A.E., Barchia I.M. (2003). Release of sterile Queensland fruit fly, *Bactrocera tryoni* (Froggatt) (Diptera: Tephritidae), at Sydney, Australia. Aust. J. Exp. Agric..

[10] Kuba H., Kohama T., Kakinohana H., Yamagishi M., Kinjo K., McPheron B.A., Steck G.J. (1996). The successful eradication programs of the melon fly in Okinawa. Fruit Fly Pests: A World Assessment of Their Biology and Management.

[11] František M., Vreysen M.J.B. (2019). Advances and Challenges of Using the Sterile Insect Technique for the Management of Pest Lepidoptera. Insects.

[12] Cáceres C., Cayol J.P., Enkerlin W., Franz G., Hendrichs J., Robinson A.S., Barnes B.N. (2004). Comparison of Mediterranean fruit fly (*Ceratitis capitata*) (Tephritidae) bisexual and genetic sexing strains: Development, evaluation and economics. Proceedings of the 6th International Symposium on Fruit Flies of Economic Importance.

[13] Klassen W., Dyck V.A., Hendrichs J., Robinson A.S. (2005). Area-Wide Integrated Pest Management and the Sterile Insect Technique, Chapter 2.1 In Sterile Insect Technique: Principles and Practice in Area-Wide Integrated Pest. Management.

[14] Hendrichs J., Franz G., Rendon P. (1995). Increased effectiveness and applicability of the sterile insect technique through male-only releases for control of Mediterranean fruit flies during fruiting seasons. J. Appl. Entomol..

[15] Franz G., Kerremans P. (1992). Radiation induced chromosome aberrations for the genetic analysis and manipulation of the Mediterranean fruit fly, Ceratitis capitata. Proceedings of the International Symposium on Management of Insect Pests. Nuclear and Related Molecular and Genetic Techniques.

[16] Franz G., Dyck V.A., Hendrichs J., Robinson A.S. (2005). Genetic sexing strains in Mediterranean fruit fly, an example for other species amenable to largescale rearing for the sterile insect technique. Sterile Insect Technique Principles and Practice in Area-Wide Integrated Pest Management.

[17] Robinson A.S., Van Heemert C. (1982). Ceratitis capitata—A suitable case for genetic sexing. Genetica.

[18] Rössler Y. (1979). Automated sexing of *Ceratitis capitata* [Dip.: Tephritidae]: The development of strains with inherited, sex-limited pupal color dimorphism. Entomophaga.

[19] Franz G., Bourtzis K., Cáceres C., Dyck V.A., Hendrichs J., Robinson A.S. (2021). Practical and Operational Genetic Sexing Systems Based on Classical Genetic Approaches in Fruit Flies, an Example for Other Species Amenable to Large-Scale Rearing for the Sterile Insect Technique. Sterile Insect Technique Principles and Practice in Area-Wide Integrated Pest Management.

[20] Fisher K., Caceres C., Rendón P., Tan K.H. (2000). A filter rearing system for mass reared sexing strains of Mediterranean fruit fly (Diptera: Tephritidae). Area-Wide Control of Fruit Flies and Other Pests.

[21] Franz G., Gencheva E., Kerremans P. (1994). Improved stability of genetic sex-separation strains for the Mediterranean fruit-fly, Ceratitis capitata. Genome.

[22] Zepeda-Cisneros C.S., Meza-Hernández J.S., García-Martínez V., Ibañez-Palacios J., Zacharopoulou A., Franz G. (2014). Development, genetic and cytogenetic analyses of genetic sexing strains of the Mexican fruit fly. *Anastrepha ludens* Loew (Diptera: Tephritidae). BMC Genet..

[23] Zepeda-Cisneros C.S., Montoya P., Toledo J., Hernández E. (2010). Desarrollo de cepas de sexado genético. Moscas de la Fruta: Fundamentos y Procedimientos para su Manejo.

[24] Braga R., Caceres C., Islam A., Wornoayporn V., Enkerlin W. (2006). Diets based on soybean protein for Mediterranean fruit fly. Pesq. Agropec. Bras..

[25] Orozco-Dávila D., Quintero-Fong L., Hernández E., Solís E., Artiaga T., Hernández R., Ortega C., Montoya P. (2017). Mass rearing and sterile insect releases for the control of Anastrepha spp. pests in Mexico—A review. Entomol. Exp. Appl..

[26] FAO/IAEA/USDA (2014). Product Quality Control for Sterile Mass-Reared and Released Tephritid Fruit Flies, Version 6.0. 2014.

[27] Resilva S.S., Hernandez E., Obra G.B. (2018). Radiation Sterilization of Mexican Fruit Fly *Anastrepha ludens* (Loew) Based on Pupal Eye Color. Philipp. J. Crop. Sci..

[28] Zacharopoulou A. (1987). Cytogenetic analysis of mitotic and salivary gland chromosomes in the medfly C. capitata. Genome.

[29] Zacharopoulou A. (1990). Polytene chromosome maps in the medfly C. capitata. Genome.

[30] García-Martinez V., Hernandez-Ortiz E., Zepeta Cisneros C.S., Robinson A.S., Zacharopoulou A., Franz G. (2009). Mitotic and polytene chromosome analysis in the Mexican fruit fly, *Anastrepha ludens* (Loew) (Diptera: Tephritidae). Genome.

[31] Balzarini M.G., Gonzalez L., Tablada M., Casanoves F., Di Rienzo J.A., Robledo C.W. (2008). InfoSat Statistical Software: Manual del Usuario.

[32] Orozco-Dávila D., Adriano-Amaya M., Quintero-Fong L., Salvador-Figueroa M. (2015). Sterility and Sexual Competitiveness of Tapachula-7 *Anastrepha ludens* Males Irradiated at Different Doses. PLoS ONE.

[33] Rendón P., McInnis D.O., Lance D., Stewart J. (2004). Medfly (Diptera: Tephritidae) genetic sexing: Large-scale field comparison of males-only and bisexual sterile fly releases in Guatemala. J. Econ. Entomol..

[34] Ramírez-Santos E., Rendón P., Ruiz-Montoya L., Toledo J., Liedo P. (2017). Effect of Irradiation Doses on Sterility and Biological Security in a Genetically Modified Strain of the Mediterranean Fruit Fly (Diptera: Tephritidae). J. Econ. Entomol..

[35] Hendrichs J., Robinson A.S., Cayol J.P., Enkerlin W. (2002). Medfly area wide sterile insect technique programmes for prevention, suppression or eradication: The importance of mating behavior studies. Fla. Entomol..

